# Hospital Mortality in the United States following Acute Kidney Injury

**DOI:** 10.1155/2016/4278579

**Published:** 2016-06-08

**Authors:** Jeremiah R. Brown, Michael E. Rezaee, Emily J. Marshall, Michael E. Matheny

**Affiliations:** ^1^The Dartmouth Institute for Health Policy and Clinical Practice, Geisel School of Medicine, Lebanon, NH 03756, USA; ^2^Department of Medicine, Dartmouth-Hitchcock Medical Center, Lebanon, NH 03756, USA; ^3^Department of Community and Family Medicine, Lebanon, NH 03756, USA; ^4^Oakland University William Beaumont School of Medicine, Rochester, MI 48309, USA; ^5^Geriatrics Research Education & Clinical Center (GRECC), Tennessee Valley Healthcare System (TVHS), Veterans Health Administration, Nashville, TN 37232, USA; ^6^Division of General Internal Medicine, Department of Medicine, Vanderbilt University School of Medicine, Nashville, TN 37232, USA; ^7^Department of Biomedical Informatics, Vanderbilt University School of Medicine, Nashville, TN 37232, USA; ^8^Department of Biostatistics, Vanderbilt University School of Medicine, Nashville, TN 37232, USA

## Abstract

Acute kidney injury (AKI) is a common reason for hospital admission and complication of many inpatient procedures. The temporal incidence of AKI and the association of AKI admissions with in-hospital mortality are a growing problem in the world today. In this review, we discuss the epidemiology of AKI and its association with in-hospital mortality in the United States. AKI has been growing at a rate of 14% per year since 2001. However, the in-hospital mortality associated with AKI has been on the decline starting with 21.9% in 2001 to 9.1 in 2011, even though the number of AKI-related in-hospital deaths increased almost twofold from 147,943 to 285,768 deaths. We discuss the importance of the 71% reduction in AKI-related mortality among hospitalized patients in the United States and draw on the discussion of whether or not this is a phenomenon of hospital billing (coding) or improvements to the management of AKI.

## 1. Introduction

Acute kidney injury (AKI) is a serious condition characterized by a sudden decline in kidney function, leading to inept excretion of nitrogenous waste from the body and deficient serum volume and electrolyte regulation. Common life-threatening complications of AKI include volume overload, hyperkalemia, acidosis, and uremia [1]. Acute tubular necrosis (ATN) caused by ischemia, exposure to nephrotoxic agents (e.g., medications and contrast media), or sepsis is the leading cause of AKI among hospitalized patients in the US [2]. Patients at risk for AKI include those with advanced age, diabetes mellitus, heart failure, liver failure, chronic kidney disease, hypotension, and sepsis. Patients who undergo cardiac/vascular surgery, organ transplantation, and mechanical ventilation or who are exposed to contrast media, nonsteroidal-inflammatory drugs (NSAIDs), antimicrobial drugs, or chemotherapeutic agents commonly experience AKI as a complicating condition [3].

According to various reports, AKI occurs in anywhere from five to twenty percent of hospitalizations in the US [4, 5]. Patients that experience AKI are at increased risk for adverse health outcomes such as end stage renal disease (ESRD), pulmonary complications, and cardiovascular events [6–9]. In addition, prior research has found that AKI patients have greater odds of all-cause mortality, increased length of stay, and an additional hospital cost of approximately $7,500 [7, 10]. AKI severity is positively associated with patient morbidity and mortality [11, 12].

The patient characteristics, procedures, and nephrotoxic agents associated with increased risk of AKI are on the rise in the United States. For example, the US population is aging and has experienced stark increases in the incidences of diabetes, heart failure, and sepsis over the last few decades [13–16]. Additionally, the utilization of procedures requiring nephrotoxic contrast media, such as cardiac catheterization, computerized tomography scans, and peripheral angiograms, as well as the use of antimicrobial drugs and NSAIDs, has grown tremendously over the same time period [2, 17–22]. Since patients developing AKI without requiring dialysis have an increased risk of in-hospital and long-term mortality, we have sought to evaluate the national trends in AKI admissions and hospital mortality [23–25].

Due to the large increase in the prevalence of risk factors for AKI, we hypothesized that the incidence of AKI has correspondingly increased among hospitalized patients. Using a nationally representative sample, we determined the incidence and associated in-hospital mortality of AKI among hospitalized patients in the US from 2001 to 2011.

## 2. Methods

### 2.1. Study Population

We extracted discharge data from 2001 to 2011 from the Healthcare Cost and Utilization Project's (HCUP) National Inpatient Sample (NIS). The NIS is a nationally representative, stratified sample of approximately 20 percent of community hospitals in the US each year. It is the largest deidentified US all-payer inpatient database publically available for research purposes. In 2001, 986 hospitals in 33 states contributed over 7.4 million discharge records to the NIS. By 2011, the NIS grew to 1,049 hospitals in 46 states and over 8 million discharge records. The committee for the Protection of Human Subjects at Dartmouth College waived required approval for this study as a publically available limited dataset.

We identified cases of AKI using the following ICD-9-CM codes for acute renal failure: 584.5 (with tubular necrosis), 584.6 (with lesion of renal cortical necrosis), 584.7 (with lesion of renal medullary necrosis), 584.8 (other specified pathologic lesions in kidney), or 584.9 (unspecified). The final cohort included 18,870,662 admissions.

We used adjusted analyses to account for comorbid conditions, age, sex, and hospital utilization practices, such as annual median length of stay and the proportion of patients discharged to skilled nursing facilities (SNF). Congestive heart failure, pulmonary circulation disorders, peripheral vascular disease, hypertension, neurological disorders, chronic pulmonary disease, diabetes without chronic complications, diabetes with chronic complications, obesity, fluid and electrolyte disorders, blood loss anemia, and deficiency anemias were identified using NIS comorbidity indicators. We also identified comorbid sepsis (038.x, 112.5, 112.81, 020.2, 790.7, 785.59). Specific ICD-9-CM codes used to identify comorbid conditions are available for review elsewhere [26].

### 2.2. Statistical Analysis

We calculated population incidence rates for AKI in the US from 2001 to 2011 by dividing the number of discharges with AKI by the US population in each year, using estimates from the US Census Bureau [27, 28]. Incidence estimates were stratified by sex and age for subgroup investigation. With data from 2001 as a reference, we used multiple logistic regression models to determine the odds of AKI and corresponding in-hospital mortality for each year included in the study. Models were adjusted for age, sex, race, congestive heart failure, pulmonary circulation disorders, peripheral vascular disease, hypertension, other neurological disorders, chronic pulmonary disease, diabetes without chronic complications, diabetes with chronic complications, obesity, fluid and electrolyte disorders, blood loss anemia, deficiency anemias, sepsis, and cardiac catheterization. Population attributable risk of in-hospital death associated AKI was calculated to approximate the proportion of patient deaths that could have been prevented if AKI was avoided. All data was analyzed using Stata 11.2 (StataCorp, College Station, TX) and weighted at the discharge level to account for the NIS sampling scheme.

## 3. Results

We identified 18,870,662 hospitalizations associated with AKI from 2001 to 2011. Over this period, significant differences were observed in all AKI patient characteristics except for sex, age, chronic pulmonary disease, and diabetes with chronic complications. Comorbid hypertension (22.9% to 61.2%), obesity (2.9% to 14.1%), and deficiency anemias (20.2% to 35.9%) increased the most for AKI patients from 2001 to 2011. Complete annual characteristics for patients with AKI are displayed in Table 1.

### 3.1. Acute Kidney Injury

**Figure 1:**
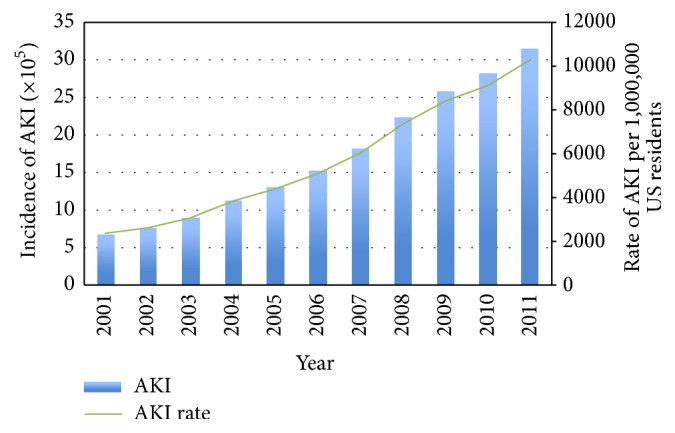
Population incidence of acute kidney injury in the United States, 2001 to 2011.

**Figure 2:**
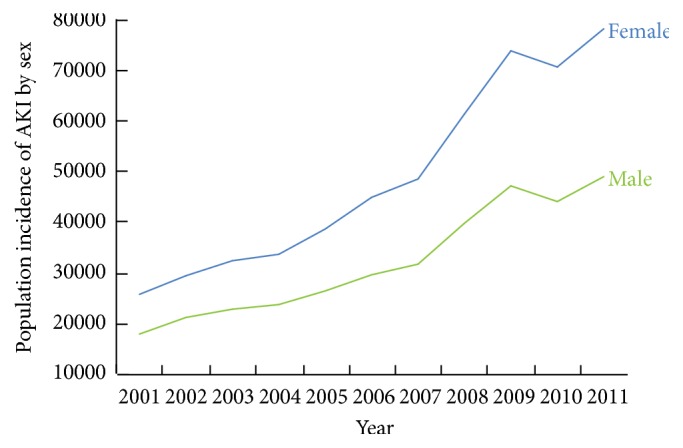
Population incidence of acute kidney injury by sex in the United States, 2001 to 2011.

**Figure 3:**
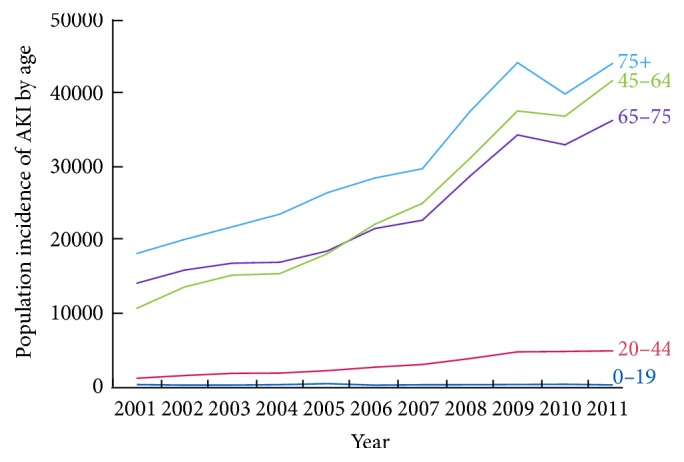
Population incidence of acute kidney injury by age group in the United States, 2001 to 2011.

We identified 18,870,662 hospitalizations associated with an AKI diagnosis over the study period. The number of cases of AKI grew from 674,845 in 2001 to 3,151,937 in 2011, an almost fivefold increase in the incidence of AKI in the US (Figure 1). Compared to 2001, the odds of AKI increasing per year in 2002 to 2011 were 1.18 (95% CI: 1.17–1.18) in unadjusted analyses (Table 2). After adjustment for patient and hospital characteristics the odds of AKI increasing per year in 2002 to 2011 compared to 2001 were 1.14 (95% CI: 1.13–1.15). Subgroup analyses revealed that men and women experienced a similar increasing pattern of AKI incidence over the study period (Figure 2). Similar patterns of AKI incidence were also found among age groups, although the highest increase in AKI incidence was observed among patients greater than 75 years of age (Figure 3).

In-hospital mortality for AKI patients decreased from 21.9% in 2001 to 9.1% in 2011. Likewise, the odds of in-hospital mortality for AKI patients in 2011 compared to 2001 were significantly reduced (unadjusted OR 0.36, 95% CI: 0.34 to 0.37; adjusted OR 0.29, 95% CI: 0.28 to 0.30; Table 3). Despite declining mortality estimates, the number of AKI patients experiencing in-hospital mortality increased twofold over the study period, from 147,943 to 285,744. In addition, the population attributable risk of death associated with AKI increased from 19.2% to 35.5% from 2001 to 2011 (Table 4).

## 4. Discussion

With this nationally representative study of hospitalizations in the US, we are the first to show that while AKI has increased, hospital mortality rates for AKI have decreased significantly from 2001 to 2011. The incidence of AKI increased fivefold over the study period. While we found a downward trend in the proportion of AKI patients with hospital mortality, the raw number of deaths and attributable population risk of death for AKI increased significantly from 2001 to 2011. By 2011, AKI was present in over 35% of all in-hospital death cases, a 16.3% increase since the beginning of the study period. Our findings demonstrate both the clinical and public health significance of AKI in the US, and the immense opportunity that exists to prevent and manage these life-threatening complications.

Our foremost contribution is to report on the growing incidence of AKI in the United States from 2001 to 2011 and to demonstrate that while AKI admissions are on the rise, hospital mortality for AKI has declined significantly. Hsu and colleagues did not study the epidemiology of nondialysis-requiring AKI due to concerns about the validity of ICD-9-CM codes without the use of companion dialysis codes to correctly identify cases [27, 29]. However, as discussed in our limitations section, it is becoming increasingly more common for studies to leverage AKI ICD-9-CM codes in claims data without dialysis as a companion code [30–32]. As a result, we were able to observe a significant increase in AKI incidence and associated in-hospital mortality among patients in our study.

The early 2000s saw the first investigations into the incidence of acute renal failure and associated in-hospital mortality. These studies expressed their findings in terms of either cases per hospitalization or intensive care unit days, making the results difficult to interpret and generalize across providers, as the number of admissions and length of stay vary between hospitals [2, 11, 33]. More recent studies have taken a more population-based approach, estimating the population incidence of AKI to be 2,000–3,000 per million people per year [27, 34, 35]. To date, only Hsu and colleagues have investigated the temporal incidence of any kind of AKI in the US (in their case, dialysis-requiring AKI (AKI-D) specifically) [27, 36]. Our analysis now provides the most up-to-date and comprehensive description of AKI and related hospital mortality epidemiology in the US. The observed increase in the incidence of AKI in our study was likely driven by the increasing commonality of patient risk factors for AKI in the US population, as well as the more frequent use of procedures and medications known to cause renal damage. However, it is important to note that the observed increase in the incidence of AKI may be due to improved recognition of AKI and coding for smaller degrees of AKI. The availability of diagnostic criteria for AKI (e.g., RIFLE) and growing provider awareness, regarding the relationship between serum creatinine and renal impairment, may have led to increased rates of AKI diagnosis over the study period [37–39]. Though unlikely, inappropriate ICD-9-CM coding practice could also have led to increased incidence estimates. In order to determine if the increase of AKI incidence is true and not a consequence of increased AKI identification through ICD-9-CM codes, we have measured AKI-D trends over the same time period in a different study [40]. AKI-D is more likely to be defined consistently over the years, and it still experiences an upward trend from 2001 to 2011, suggesting that increasing AKI incidence is not solely a result of inappropriate ICD-9-CM coding [40].

The strengths of our study include the use of a large nationally representative sample of hospitalizations in the US reporting on the incidence of AKI and hospital mortality. In addition, our use of population attributable risk of mortality estimates allows readers to understand both the clinical and public health significance of AKI in the US.

However, our study has several important limitations. First, as stated previously, we utilized ICD-9-CM codes to identify cases of AKI. Prior analyses have not utilized ICD-9-CM codes alone to identify cases of AKI due to concerns of validity [27, 29]. We leveraged prior research demonstrating the high specificity (97.7%) and low sensitivity (35.4%) of ICD-9-CM codes in identifying cases of AKI for this analysis [29, 41]. Second, administrative billing codes are less useful than clinical data for identifying AKI. Clinical or laboratory data, such as serum creatinine levels, would have provided the most accurate marker of renal impairment had they been available [29]. Third, the NIS is a completely deidentified dataset, making it impossible to track specific patients over time.

Although our study establishes the significance of the problem, additional research is needed on the drivers of drastic increase of AKI incidence in the US. More research is also needed to understand geographic and subgroup variation, as well as which prevention and management strategies result in the greatest decline and control of AKI among hospitalized patients.

In summary, our study demonstrates that the incidence of AKI increased significantly in the US between 2001 and 2011. The percentage of all in-hospital deaths occurring in combination with AKI rose by 16.3% over the study period. AKI represents a growing clinical and public health problem for Americans.

## Figures and Tables

**Table 1:** Characteristics of patients with acute kidney injury in the United States, 2001 to 2011.

	All years	2001	2002	2003	2004	2005	2006	2007	2008	2009	2010	2011	*P* value
*N*	18,870,662	674,845	752,472	891,353	1,125,378	1,299,289	1,522,004	1,818,011	2,236,412	2,581,314	2,817,647	3,151,937	
Female (%)	47.6	47.42	47.37	48.02	47.99	47.69	47.35	47.45	47.85	47.56	47.44	47.65	0.46
Age (median)	72	72	72	72	72	72	72	72	72	71	71	71	0.72
Age (IQR)	58–81	59–81	59–81	59–81	59–81	58–81	58–81	58–81	59–82	58–81	58–81	58–82	0.72
Length of stay (median)	6	7	7	7	7	6	6	6	6	5	5	5	<0.001
Length of stay (IQR)	3–11	4–13	4–13	4–13	3–12	3–12	3–11	3–11	3–10	3–10	3–10	3–9	<0.001
Heart failure (%)	22.78	23.68	23.99	24.42	25.22	25.37	24.75	23.65	21.43	21.18	21.06	22.04	<0.001
Pulmonary disease (%)	3.53	1.33	1.51	1.54	1.56	1.61	2.1	3.25	3.94	4.19	4.59	5.03	<0.001
Peripheral vascular disease (%)	8.94	6.34	7.2	6.88	6.85	6.73	7.42	8.37	9.43	9.59	9.36	10.89	<0.001
Hypertension (%)	52.86	22.91	26.78	44.15	45.44	46.4	49.19	48.69	53.42	56.73	58.14	61.21	<0.001
Neurological disease (%)	9.61	3.62	9.2	7.55	7.81	7.79	8.3	9.3	9.79	10.05	10.3	11.12	<0.001
Chronic pulmonary disease (%)	23.4	20.9	22.23	22.62	22.91	23.8	24.31	24.36	22.36	22.9	22.84	24.11	0.29
Diabetes (%)	23.72	17.12	18.6	19.18	19.56	19.36	21.17	22.71	24.15	25.65	25.97	27.26	<0.001
Diabetes with sequelae (%)	10.94	9.55	10.86	10.61	10.17	10.32	10.83	11.23	10.75	10.53	10.41	11.57	0.28
Obesity (%)	9.26	2.94	3.9	4.17	4.37	4.89	5.96	7.35	9.8	11.09	11.26	14.08	<0.001
Anemia (%)	30.47	20.19	22.85	23.86	23.6	23.58	26.27	28.9	32.11	32.84	32.48	35.87	<0.001
Sepsis (%)	20.8	19.31	19.87	19.65	19.88	20.35	20.58	20.55	20.87	20.52	20.58	21.12	<0.001
In-hospital mortality (%)	12.32	21.92	20.38	18.76	16.76	15.38	14.09	12.36	11.8	10.49	9.74	9.07	<0.001

**Table 2:** Crude and adjusted odds ratios for acute kidney injury in the United States, 2011 compared to 2001.

AKI				
Population at risk	Crude OR/yr	95% CI	Adjusted OR/yr	95% CI
Overall	1.18	(1.17–1.18)	1.14	(1.13–1.15)
*Age groups*				
0–19	1.10	(1.07–1.14)	1.07	(1.05–1.09)
20–44	1.17	(1.16–1.19)	1.12	(1.11–1.13)
45–64	1.18	(1.17–1.19)	1.13	(1.13–1.15)
65–74	1.17	(1.16–1.18)	1.14	(1.13–1.15)
75+	1.19	(1.18–1.19)	1.15	(1.15–1.16)
*Sex*				
Male	1.18	(1.17–1.18)	1.14	(1.13–1.15)
Female	1.18	(1.17–1.18)	1.14	(1.13–1.15)

**Table 3:** Crude and adjusted odds ratios for mortality amongst acute kidney injury and dialysis-requiring acute kidney injury in the United States, 2011 compared to 2001.

Odds of mortality following AKI				
Year	Crude OR	95% CI	Adjusted OR	95% CI
2001	Referent			
2002	0.90	(0.87–0.94)	0.92	(0.88–0.97)
2003	0.82	(0.79–0.85)	0.87	(0.83–0.91)
2004	0.72	(0.69–0.75)	0.74	(0.70–0.77)
2005	0.65	(0.62–0.68)	0.65	(0.62–0.69)
2006	0.58	(0.56–0.61)	0.60	(0.58–0.63)
2007	0.50	(0.48–0.53)	0.52	(0.49–0.55)
2008	0.48	(0.46–0.50)	0.51	(0.48–0.53)
2009	0.42	(0.40–0.44)	0.45	(0.43–0.48)
2010	0.38	(0.37–0.40)	0.42	(0.40–0.44)
2011	0.36	(0.34–0.37)	0.29	(0.28–0.30)

**Table 4:** Population attributable risk of mortality associated with acute kidney injury and dialysis-requiring acute kidney injury in the United States, 2001 through 2011.

Year	Population attributable risk of mortality following AKI
2001	19.2
2002	21.0
2003	22.4
2004	23.7
2005	24.8
2006	26.5
2007	28.6
2008	31.1
2009	33.7
2010	34.4
2011	35.5
